# Multilayer Core-Sheath Structured Nickel Wire/Copper Oxide/Cobalt Oxide Composite for Highly Sensitive Non-Enzymatic Glucose Sensor

**DOI:** 10.3390/nano15060411

**Published:** 2025-03-07

**Authors:** Yuxin Wu, Zhengwei Zhu, Xinjuan Liu, Yuhua Xue

**Affiliations:** School of Materials and Chemistry, University of Shanghai for Science and Technology, 516 Jungong Road, Shanghai 200093, China; 222203074@st.usst.edu.cn (Y.W.); zzw12342025@163.com (Z.Z.)

**Keywords:** non-enzymatic glucose sensor, core-sheath, copper oxide, cobalt oxide nanowires, nickel wire

## Abstract

The development of micro glucose sensors plays a vital role in the management and monitoring of diabetes, facilitating real-time tracking of blood glucose levels. In this paper, we developed a three-layer core-sheath microwire (NW@CuO@Co_3_O_4_) with nickel wire as the core and copper oxide and cobalt oxide nanowires as the sheath. The unique core-sheath structure of microwire enables it to have both good conductivity and excellent electrochemical catalytic activity when used as an electrode for glucose detecting. The non-enzymatic glucose sensor base on a NW@CuO@Co_3_O_4_ core-sheath wire exhibits a high sensitivity of 4053.1 μA mM^−1^ cm^−2^, a low detection limit 0.89 μM, and a short response time of less than 2 s.

## 1. Introduction

Glucose biosensors are essential in the medical field for monitoring blood glucose levels, as well as in the food industry for assessing sugar content given that high sugar intake is associated with increased cardiovascular disease risk [1,2,3]. In recent years, researchers have directed their efforts toward the development of non-enzymatic glucose sensors due to their faster response times, good stability, and lower costs [4,5]. Inorganic nanometals and metal oxides have been widely used in non-enzymatic electrochemical biosensors due to their long-term stability, high surface area, and excellent electrocatalytic activity [6,7]. However, studies involving metal oxide composites as active materials are relatively limited. Well-designed metal oxide composites can provide more active sites, significantly enhance electrocatalytic activity, facilitate electron transfer, and ultimately improve the sensor’s sensitivity.

Fiber-based sensors are ideally suited for the ongoing trend of miniaturization and smart applications, as their fiber-shaped and core-sheath structure design significantly enhances testing efficiency. Moreover, their remarkable flexibility and plasticity make them applicable across a diverse range of scenarios [8]. Zhao et al. reported a fiber-shaped biosensor based on gold fibers, which exhibited a high a sensitivity of 11.7 μA mM^−1^ cm^−2^ [9]. Carbon nanofibers have been reported for the development of an impedance sensor [10,11], and carbon nanofibers modified with aromatic diamine were used for glucose sensor with a high selectivity [12]. SnO_2_ nanofibers could be used for the development of a plasmonic resonance sensor [13]. The SnO_2_ nanofiber-based sensors could perform real-time monitoring of mammalian cells [14], and Cu_2_O-modified SnO_2_/N-C nanofibers could be used for NO_2_ detection [15]. Our group has grown gold nanomaterials on graphene fibers and used them for hydrogen peroxide and glucose detection, which showed excellent performance [16]. We also reported fiber-shaped glucose biosensors with F-doped nickel hydroxide nanorods on graphene fibers [17]. However, the preparation process of graphene fibers is relatively complicated, and the conductivity of graphene fibers is not very good.

Copper oxide (CuO) has found wide-ranging applications in photocatalysis, electrocatalysis, sensing, and antibacterial fields. This versatility is attributed to its high abundance, favorable redox potential, biocompatibility, and excellent stability. As the electrocatalytic activity of copper oxide depends on its surface properties and morphology, several researchers have employed various methods to synthesize copper oxide nanostructures that can be used for sensitive and selective detection of lactate and glucose [18,19]. The p-type copper oxide (CuO)-based photoelectric cathode, characterized by its substantial copper content, optimal bandgap, and advantageous band alignment, emerges as a promising candidate for large-scale photoelectrocatalytic water decomposition [20]. Its p-type conductivity can lead to limitations in performances under certain conditions, such as high glucose concentrations, which may cause sensitivity issues or interference from other substances. Composites of multiple metals, metal oxides, or carbon materials is one of the effective ways to solve this problem. For example, when using copper oxide/carbon layer core-shell composites as a non-enzymatic glucose sensor, they exhibited a good sensitivity of 272.6 μA mM^−1^ cm^−2^ [21]. Copper oxide/zinc oxide composites also displayed good sensitivity for glucose detection [22]. Co_3_O_4_ is a versatile inorganic compound known for its excellent electrochemical catalytic properties. Co_3_O_4_-based composites have been used as high-performance glucose sensors [23,24].

Herein, we have a rationale designed and prepared a three-layer core-sheath microwire (NW@CuO@Co_3_O_4_), with nickel wire as the core and copper oxide and cobalt oxide nanowires as the sheath, the latter of which is expected to demonstrate a synergistic effect for enhancing the performance for glucose detection. Nickel wire as the core has good conductivity and flexibility. The CuO and Co_3_O_4_ composite in the sheath layer has very good electrocatalytic activity, and the electrons generated by it can be quickly transferred to the nickel wire in the core. Finally, the NW@CuO@Co_3_O_4_ core-sheath wire was applied as the wire-shaped electrode for glucose sensing.

## 2. Experimental

### 2.1. Chemicals

Nickel sulphate (NiSO_4_·6H_2_O), boric acid (H_3_BO_3_), and copper sulphate (CuSO_4_·5H_2_O) were purchased from Shanghai Aladdin Reagent Company (Shanghai, China). Cobalt acetate tetrahydrate (C_4_H_14_CoO_8_), ammonium fluoride (NH_4_F), and urea (NH_2_CONH_2_) were purchased from Shanghai Titan Technology Co., Ltd. (Shanghai, China).

### 2.2. Preparation of Nickel Wire, Copper Oxide and Cobalt Oxide (NW@CuO@Co_3_O_4_) Composite Wire

A nickel wire with a diameter of 100 μm was first ultrasonically treated in acetone for 5 min to remove surface impurities and then washed with ionized water. The electrolyte was prepared by dissolving 0.2 M nickel sulfate (NiSO_4_·6H_2_O), 0.01 M copper sulfate (CuSO_4_·5H_2_O), and 0.1 M boric acid (H_3_BO_3_) in 50 mL DI water. The electrodeposition process took place in an electrochemical workstation with the nickel wire as the working electrode at a voltage of −0.8 V for 20 min. Then, the voltage was changed to +0.5 V and kept for another 20 min

The as-prepared wire was washed with DI water and transferred into a mixed aqueous solution containing 5 mM cobalt acetate tetrahydrate (C_4_H_14_CoO_8_), 10 mM ammonium fluoride (NH_4_F), and 25 mM urea (NH_2_CONH_2_). This aqueous solution was then placed in a Teflon-lined hydrothermal reactor and subjected to a hydrothermal reaction at 120 °C for 6 h in an oven. After completing the hydrothermal reaction, the sample was removed and washed with water. The dried wire was then placed in a tube furnace for annealing at 400 °C for 2 h to obtain the final product.

### 2.3. Characterization

Transmission electron microscope (TEM) images were captured with a JEOL 2000 microscope operating at 200 kV. Scanning electron microscopy (SEM) images were acquired using an FEI Quanta 450 FEG equipment. X-ray diffraction (XRD) analyses were carried out with an XPERT-PRO diffractometer featuring CuKα radiation (λ = 1.54 Å). X-ray photoelectron spectroscopy (XPS) spectra were recorded on a Thermo Fisher Scientific K-Alpha spectrometer with Al Ka X-ray radiation for excitation.

Prior to electrochemical testing, sensor electrode assembly was performed following the procedure illustrated in Figure S1. The NW@CuO@Co_3_O_4_ core-sheath wire was initially bonded to a copper wire using conductive silver adhesive. Subsequently, the integrated wire was encapsulated in a polyvinylidene fluoride (PVDF) tube (inner diameter of 2 mm), leaving 5 mm of the electrode outside the tube. Both ends of the PVDF tube were sealed with epoxy resin, while the copper wire could be connected to the electrochemical workstation via an electrode clamp.

Electrochemical testing was conducted on the CHI 660 Electrochemical Workstation with a three-electrode system in 0.1 M NaOH solution. An Ag/AgCl (4 M KCl) electrode and a platinum wire (diameter 0.5 mm) were used as the reference electrode and the counter electrode, respectively. The NW@CuO@Co_3_O_4_ electrode assembled as described above was used as the working electrode. For CV tests, the scan rate was 50 mV/s unless otherwise mentioned. For amperometric i–t tests, the electrolyte was stirred at 200 rpm to provide convective transport. All potentials were reported relative to the Ag/AgCl reference system.

## 3. Results and Discussion

**Figure 1:**
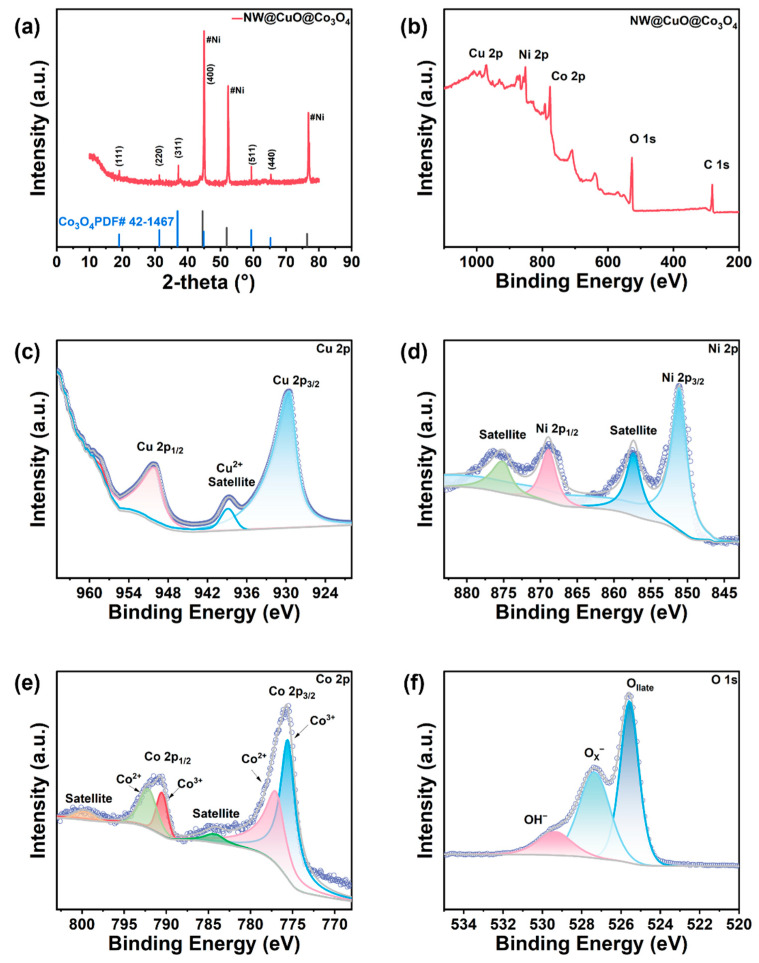
(**a**) XRD spectrum of the NW@CuO@Co_3_O_4_ electrode. XPS spectra of the NW@CuO@Co_3_O_4_ electrode, (**b**) full spectrum, (**c**) Cu 2p spectrum, (**d**) Ni 2p spectrum, (**e**) Co 2p spectrum, and (**f**) O 1s spectrum.

In order to understand the phase composition and crystal structure of the NW@CuO@Co_3_O_4_ electrode, XRD tests were conducted. As shown in Figure 1a, eight characteristic peaks were observed at 2θ values of 19.1°, 31.3°, 37.0°, 44.8°, 52.23°, 59.4°, 65.3°, and 78.01°. Based on PDF card JCPDS 42-1467, the peaks at 2θ = 19.1°, 31.3°, 37.0°, 44.8°, and 59.4° correspond to the (111), (220), (311), (400), and (511) crystal planes of Co3O4, respectively. Additionally, according to PDF card JCPDS 03-1051, the peaks at 2θ = 44.83°, 52.23°, and 78.01° correspond to the (111), (200), and (220) crystal planes of the nickel matrix, indicating that Co_3_O_4_ was successfully synthesized on the nickel wire substrate. It is worth noting that there is no obvious characteristic peak of copper oxide in the entire XRD spectrum. This is because copper oxide is covered by cobalt oxide and the content of copper oxide is relatively small, so its diffraction peak is not obvious.

Figure 1b displays the full spectrum of NW@CuO@Co_3_O_4_, where the signals for Ni, Co, O, and Cu arise from the nickel wire matrix as well as the loaded materials, CuO and Co_3_O_4_, respectively. In the high-resolution spectrum of Cu 2p, as depicted in Figure 1c, a peak at a binding energy of 929.5 eV is attributed to Cu 2p_3/2_, accompanied by a peak at 938.6 eV. The peak at 950.5 eV represents Cu 2p_1/2_, with a concomitant peak at 958.2 eV. Through the comprehensive analysis of the Cu spectrum, the difference in binding energy between Cu 2p_1/2_ and Cu 2p_3/2_ is found to be 21 eV, indicating that the copper in the NW@CuO@Co_3_O_4_ electrode is in the divalent state [25]. This indicated that the copper deposited on electrode after alloying and dealloying was oxidized to generate CuO during the subsequent hydrothermal process and high-temperature annealing process. Figure 1d presents the Ni 2p spectrum, where peaks 869.2 eV and 857.0 eV correspond to Ni 2p_1/2_ and Ni 2p_3/2_, respectively, along with characteristic peaks at 857.0 eV and 869.2 eV. These peaks are attributed to Ni^2+^, indicating the presence of divalent nickel in the NW@CuO@Co_3_O_4_ electrode. It is possible that part of the nickel was oxidized during the electrode preparation process.

Figure 1e shows the high-resolution spectrum of Co 2p. The peaks at 790.8 eV and 775.8 eV are associated with Co 2p_1/2_ and Co 2p_3/2_, respectively. Additionally, the binding energies at 800.2 eV and 784.3 eV are linked to the satellite peaks of Co 2p_1/2_ and Co 2p_3/2_. The Co 2p_1/2_ peak can be decomposed into two components at 792.3 eV and 790.6 eV, representing Co^2+^ and Co^3+^, respectively. Similarly, the Co 2p_3/2_ peak at 775.8 eV can be decomposed into two peaks at 777.3 eV and 775.5 eV, corresponding to Co^2+^ and Co^3+^, respectively. This confirms that the Co element in the NW@CuO@Co_3_O_4_ electrode exists as a mixture of divalent and trivalent states [26].

The O1s XPS spectrum (Figure 1f) is deconvoluted into three characteristic peaks at binding energies of 525.6 eV, 527.4 eV, and 529.4 eV. The peak at 525.6 eV corresponds to lattice oxygen within the NW@CuO@Co_3_O_4_ electrode, while the peak at 527.4 eV is associated with oxygen that occupies lattice vacancies in NW@CuO@Co_3_O_4_. The peak at 529.4 eV represents physically and chemically adsorbed oxygen on the electrode surface.

**Figure 2:**
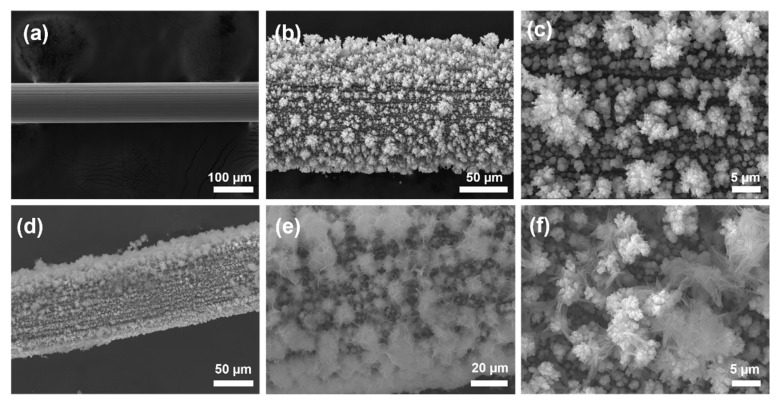
(**a**) SEM image of pure nickel wire. (**b**,**c**) SEM image of nickel wire after “alloy/de-alloy” treatment. (**d**–**f**) SEM image of NW@CuO@Co_3_O_4_ electrode.

Figure 2a illustrates that the surface of the pure nickel wire is relatively smooth, with a diameter of approximately 100 μm. As shown in Figure 2b,c, after treating the nickel wire using the alloy/de-alloy method, a significant amount of copper nanoparticles were observed on its surface. These nanoparticles vary in size and are stacked upon one another, resulting in an uneven surface on the previously smooth nickel wire. Figure 2d reveals that a layer of Co_3_O_4_ was uniformly coated to the treated nickel wire through a hydrothermal process. The entire NW@CuO@Co_3_O_4_ composite wire has an approximate diameter of 130 μm, with a coating thickness of about 15 μm. As shown in Figure 2e, the Co_3_O_4_ particles are clustered and tightly connected to the copper nanoparticles formed during the initial step of alloy dealloying electrodeposition, which may facilitate the formation of a core–shell structure between these two metal oxides. The high-resolution SEM image in Figure 2f depicts a single cluster resembling a dandelion, consisting of multiple nanowires approximately 10 µm in length. This structure increases the specific surface area of the material, promoting redox reactions between the material and glucose.

**Figure 3:**
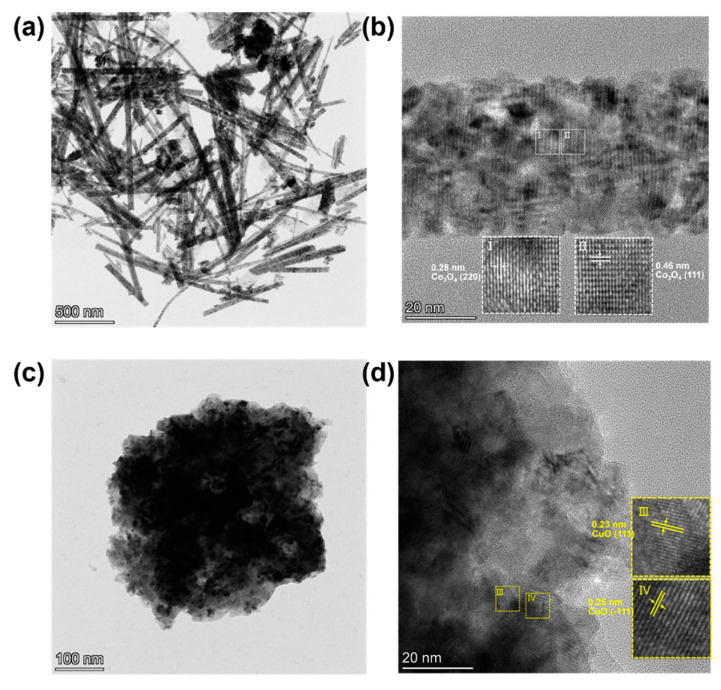
(**a**) TEM image of CuO–Co_3_O_4_ composite. (**b**) TEM image of Co_3_O_4_ nanowires, (**c**,**d**) TEM images of CuO nanoparticles.

Figure 3 presents the transmission electron microscope (TEM) image of NW@CuO@Co_3_O_4_. As shown in Figure 3a, the mixture of nanoparticles and nanowires is observed, which is consistent with the scanning electron microscope (SEM) image shown in Figure 2d. The high-magnification TEM image in Figure 3b reveals that the two lattice spacings correspond to the (220) and (111) crystal planes of Co_3_O_4_. Figure 3c shows the CuO nanoparticles with a diameter of approximately 500 nm. Figure 3d presents the high-resolution TEM image of a CuO nanoparticle, revealing two lattice spacings of 0.23 nm and 0.25 nm that are attributed to the (111) and (−111) crystal planes of CuO, respectively.

**Figure 4:**
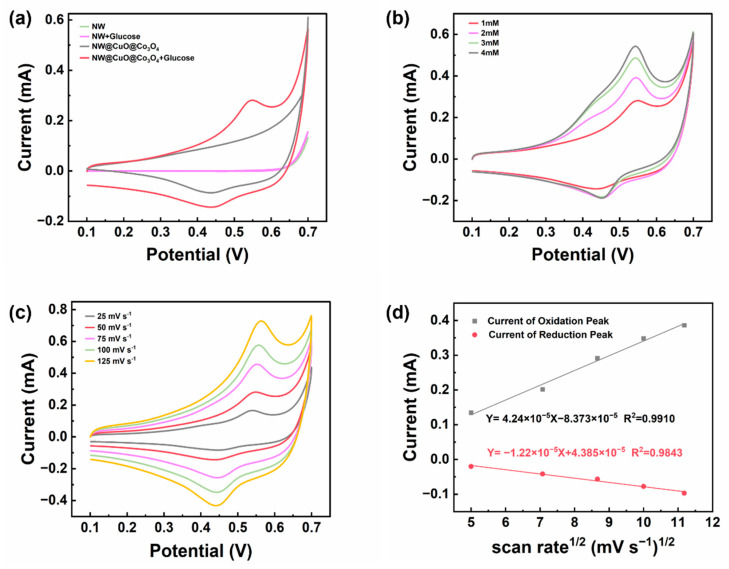
(**a**) Cyclic voltammograms of pure nickel wire electrode and NW@CuO@Co_3_O_4_ electrode at 50 mV s^−1^ in 0.1 M NaOH solution with and without 1mM glucose added. (**b**) Cyclic voltammograms of NW@CuO@Co_3_O_4_ electrode at 50 mV s^−1^ in different 0.1M NaOH solutions with different concentrations of glucose added. (**c**) Cyclic voltammograms of NW@CuO@Co_3_O_4_ electrode in 0.1 M NaOH at different scan rates (25~125 mv s^−1^). (**d**) Linear fitting diagram of cyclic voltammograms of oxidation peak current and reduction peak current at different scan rates and the half of the scan rate.

To assess the electrochemical performance of the NW@CuO@Co_3_O_4_ electrode, we tested both a pure nickel wire and the NW@CuO@Co_3_O_4_ electrode in a 0.1 M NaOH solution. Cyclic voltammetry tests were recorded in the electrolyte without and with the addition of 1 mM glucose. As depicted in Figure 4a, the results indicated that, regardless of glucose presence, the pure nickel wire did not exhibit significant redox peaks in the alkaline solution. This suggests that the nickel wire does not catalyze glucose in this system. Conversely, the NW@CuO@Co_3_O_4_ electrode demonstrated distinct redox peaks in alkaline solution. Notably, the peak current of these redox peaks increased significantly after the addition of glucose, indicating the occurrence of a redox reaction between the electrode and glucose, resulting in the generation of an additional current.

The NW@CuO@Co_3_O_4_ electrode participates in a redox reaction in alkaline solution, producing CoOOH. This CoOOH subsequently reacts further in the alkaline environment to form CoO_2_. Upon the addition of glucose, the equilibrium of this reaction is disrupted: CoO_2_ is consumed, glucose is oxidized, and CoO_2_ is reduced back to CoOOH. This process re-establishes a new equilibrium within the entire reaction system, resulting in an increase in the redox peak current. The specific reaction equations are as follows [27]:

$${Co}_{3}O_{4}+{OH}^{-}+H_{2}O↔3CoOOH+e^{-}$$

$$CoOOH+{OH}^{-}↔CoO_{2}+H_{2}O+e^{-}$$

$$2CoO_{2}+C_{6}H_{12}O_{6}\to C_{6}H_{10}O_{6}+2CoOOH$$

At the same time, copper oxide also participates in the catalysis of glucose in the following equations [28]:

$$CuO+{OH}^{-}\to CuOOH+2e^{-}$$

$$2CuOOH+glucose(C_{6}H_{12}O_{6})\to 2CuO+H_{2}O+Glucolactone(C_{6}H_{10}O_{6})$$

Figure 4b presents the CV curves at varying glucose concentrations. It is clear that in an alkaline solution, as glucose concentration increases, the oxidation peak current on the cyclic voltammogram rises accordingly, whereas the reduction peak current exhibits minimal variation. Furthermore, these results demonstrate that the NW@CuO@Co_3_O_4_ electrode possesses excellent catalytic oxidation properties toward glucose, generating distinct peak currents in response to varying concentrations of glucose in alkaline solutions.

Figure 4c displays CV curves at varying scan rates in a solution containing 1 mM glucose and 0.1 M NaOH. As the scan rate increases, the peak oxidation current rises and shifts to the right, while the peak reduction current simultaneously increases and shifts to the left. To clarify the relationship between peak currents and scan rates, a linear fit between the square root of half the scan rate and the peak current is provided in Figure 4d. The linear correlation coefficient for the peak oxidation current relative to the square root of half the scan rate is 0.9910, while for the peak reduction current, it is 0.9843. This linear relationship indicates that the entire reaction system is diffusion-controlled.

**Figure 5:**
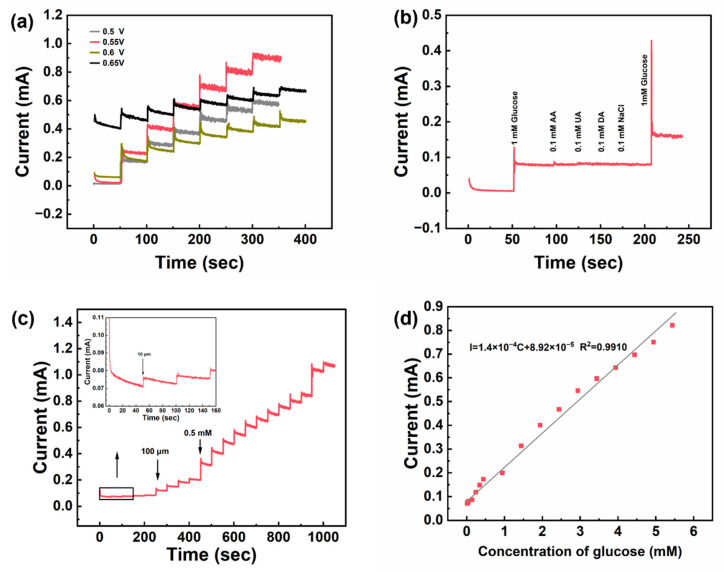
Glucose detection performance of NW@CuO@Co_3_O_4_ electrode (**a**) Current versus time curves of adding 0.5 mM glucose six times at 50 s intervals to 0.1 M NaOH solution at different voltages (0.5~0.65 V). (**b**) Selectivity curve after adding 1 mM glucose, ascorbic acid (AA), dopamine hydrochloride (UA), uric acid (DA), and glucose to 0.1 M NaOH solution in sequence. (**c**) Amperometric response curve of adding different concentrations of glucose in 0.1 M NaOH solution in sequence at 0.55 V. (**d**) Linear fitting diagram of current and glucose concentration in alkaline solution.

The operating voltage of the glucose sensor significantly influences its functional performance. Selecting an optimal operating voltage can enhance the efficiency of glucose catalytic oxidation, thereby ensuring optimal sensitivity and stability of the sensor. Cyclic voltammograms indicate that the oxidation peak occurs in a voltage range of 0.5 V to 0.65 V. A glucose solution was continuously added to a 0.1 M NaOH solution at voltages of 0.5 V, 0.55 V, 0.6 V, and 0.65 V, as illustrated in Figure 5a. An immediate response current was generated upon the addition of glucose, confirming that the NW@CuO@Co_3_O_4_ electrode exhibits a robust oxidative response to glucose. Within the voltage range of 0.5 V to 0.6 V, the response current increases with the rising voltage. However, at 0.65 V, the initial background current increases significantly. Following the addition of glucose, the increase in current does not surpass that observed at 0.55 V, and the response current becomes unstable with the gradual addition of glucose. Therefore, based on a comprehensive analysis of the data, 0.55 V is identified as the optimal operating voltage for this electrode.

Selectivity is a vital parameter in assessing the performance of the glucose sensor. Various other interfering substances may be present in the glucose solution environment, such as the structural analog of glucose, ascorbic acid (AA), dopamine hydrochloride (DA), and uric acid (UA). The principle of the glucose sensor is to convert oxidized glucose into a response current for characterizing glucose concentration. Consequently, it is essential for the sensor to selectively identify glucose molecules amidst the presence of other substances. To evaluate the sensor’s specificity, a series of tests were conducted with 0.5 mM glucose added to 0.1 M NaOH at 0.55 V, alongside AA, DA, UA, and NaCl. As shown in Figure 5b. The data reveal that a response current is generated upon the addition of glucose, while no significant response current is observed with other interfering substances. Moreover, the introduction of glucose after adding the interfering substances still elicits a response current, indicating that the glucose sensor does not adsorb these substances onto the electrode surface, thereby preserving its selective catalytic activity toward glucose. Based on these findings, the NW@CuO@Co_3_O_4_ electrode demonstrates excellent selectivity in glucose sensing.

To examine the relationship between glucose concentration and response current, glucose solutions of 0.01 mM, 0.1 mM, and 0.5 mM were sequentially added at an operating voltage of 0.55 V in 0.1 M NaOH solution, as shown in Figure 5c. The figure demonstrates that as the concentration of added glucose increases, the response current also escalates. Furthermore, as the concentration difference of the added glucose increases, the difference in response current correspondingly increases. With each addition of glucose, the response current exhibits a slight incremental rise until it becomes unstable, indicating that the sensor has reached its detection limit. A linear regression analysis of glucose concentration versus response current is presented in Figure 5d. The fitting relationship for glucose concentrations ranges from 0.01 mM to 5.44 mM. The linear correlation coefficient R2 is 0.991, the sensitivity of the NW@CuO@Co_3_O_4_ electrode is calculated to be 4053.1 μA·mM^−1^·cm^−2^, the current response time is less than 2 S, and the lowest detection limit is about 0.89 μM.

**Figure 6:**
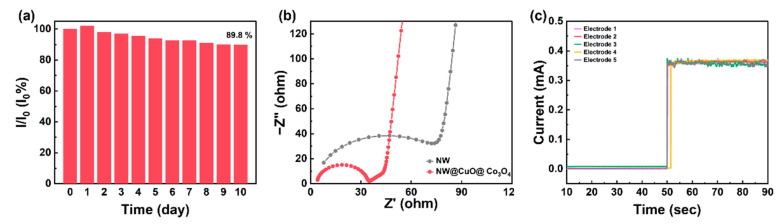
(**a**) Stability of NW@CuO@Co_3_O_4_ glucose sensors in 0.1 mM glucose solution. (**b**) EIS curves of NW and NW@CuO@Co_3_O_4_. (**c**) Current responses of five NW@CuO@Co_3_O_4_ electrodes in 0.1 M NaOH with 2 mM glucose at 0.55 V.

The long-term stability of the microelectrode was assessed by monitoring its current response in a mixed glucose solution containing 0.1 M NaOH and 2 mM glucose for 10 days. The testing method involves evaluating the current response of the same electrode after each day of its 100th use. As illustrated in Figure 6a, after 10 days (equating to 1000 reuses), the current response of the electrode maintained 89.8% of its initial value. Prior to testing, the electrode was stored in air at approximately 20 °C and 50% relative humidity.

Electrochemical impedance spectroscopy was conducted on the core-sheath microelectrode (NW@CuO@Co_3_O_4_) in a mixed glucose solution of 0.1 M NaOH and 2 mM glucose. As depicted in Figure 6b, the Nyquist plot features a semicircle in the high-frequency region and a linear component in the low-frequency region. The charge transfer resistance (Rct) can be determined by the diameter of the semicircle in the plot. The Rct for NW@CuO@Co_3_O_4_ microelectrode is 18.98 Ω and the series resistance (Rs) of this electrode is 3.458 Ω, which are much smaller than those of unmodified pure nickel wire (NW) electrode (Rct: 76.93 Ω and Rs: 4.29 Ω). The Rct decreases as the glucose concentration increases (Figure S2). The copper oxide and cobalt oxide in the sheath significantly reduce the resistance of the wire electrode and thus enhance the electron transmission efficiency of the electrode. The performance of the unmodified pure nickel wire is shown in Figure S3. Therefore, the performance of the NW@CuO@Co_3_O_4_ electrode for glucose detecting is much better than unmodified pure nickel wire and some glucose sensors in recent literature (Table 1).

In order to study its repeatability, the amperometric response of five electrodes towards 2 mM glucose was tested in 0.1 M NaOH (Figure 6c). A relative standard deviation (R.S.D.) of five electrodes was calculated to be 3.12%, confirming the good reproducibility of the NW@CuO@Co_3_O_4_ electrode.

A real sample recovery test was conducted using 50% (10 g/20 mL) glucose drinks sold on the market. The i–t method was utilized for testing and a linear fit was performed on the current density and glucose concentration. The glucose concentration was calculated and shown in Table S1 and Figure S4. The recoveries were in the range of 97–103.5%, illustrating the core-sheath wire electrode has outstanding reliability for glucose determination.

## 4. Conclusions

In summary, we designed and synthesized a three-layer core-sheath microwire (NW@CuO@Co_3_O_4_), with nickel wire as the core and copper oxide and cobalt oxide nanowires as the sheath. The synergistic effect of copper oxide nanoparticles and cobalt oxide nanowires enables it to have very high electrochemical activity, while the core-sheath structure gives the electrode very low resistances, including low Rct and Rs. The glucose sensor based on NW@CuO@Co_3_O_4_ electrode has good performance, with a linear range of 0.01~5.44 mM and a detection limit of 0.89 μM. The NW@CuO@Co_3_O_4_-based glucose sensor also exhibits good selectivity and stability.

## Figures and Tables

**Table 1:** Comparison of performances of our electrode with other metal oxide-flexible glucose sensors.

Sensitive Materials	Operating Potential	Linear Interval (Up to, mM)	Sensitivity (μA· mM^−1^·cm^−2^)	Detection Limit (μM)	Ref
CuO biscuits/SPCE	0.5 V	4.03	308.71	0.1	[29]
Ni(OH)F@GF	0.55 V	7.66	595.3	1	[17]
CuCo_2_O_4_	0.55 V	7.9	400	0.75	[30]
Co(NO_3_)_2_@rGO	0.45 V	4	1210	1.4	[31]
Zn-Co-OH/3D NF	0.6 V	3.53	6100	8.33	[32]
NW	0.55 V	0.5	270	45.2	this work
NW@CuO@Co_3_O_4_	0.55 V	5.44	4053	0.89	this work

## Data Availability

No new data were created or analyzed in this study.

## Supplementary Materials

The following supporting information can be downloaded at: https://www.mdpi.com/article/10.3390/nano15060411/s1, Figure S1. (a) Schematic illustration of assembly for the NW@CuO@Co_3_O_4_ electrode. (b,c) Photographs of bare nickel microwire, NW@CuO@Co_3_O_4_ microwire and final sensor electrode. Figure S2. EIS of NW@CuO@Co_3_O_4_ microwire in 0.1 M NaOH with different concentration glucose. Figure S3. (a) Bare NW Amperometric response curve of adding different concentrations of glucose in 0.1 M NaOH solution in sequence at 0.55 V (b) Linear fitting diagram of current. Figure S4. Determination of glucose concentration in drinks by NW@CuO@Co_3_O_4_ electrode. Table S1. Determination of glucose concentration in drinks by NW@CuO@Co_3_O_4_ electrode.
