# Supplementary material for: Multilayer Core-Sheath Structured Nickel Wire/Copper Oxide/Cobalt Oxide Composite for Highly Sensitive Non-Enzymatic Glucose Sensor
Source: Nanomaterials (Basel). 2025 Mar 7;15(6):411. doi: 10.3390/nano15060411 (PMC11946131; doi:10.3390/nano15060411)
Supplement: Supplementary file 1 [file nanomaterials-15-00411-s001.zip › nanomaterials-3465073-supplementary.pdf]

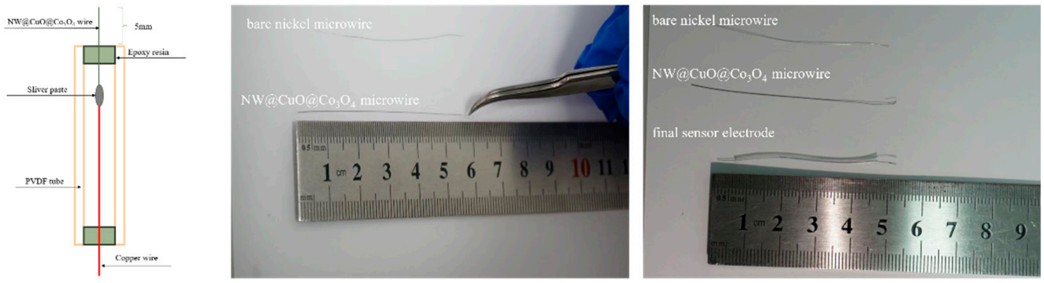

Figure S1 (a) Schematic illustration of assembly for the NW@CuO@Co_3O_4 electrode. (b, c) Photographs of bare nickel microwire, NW@CuO@Co_3O_4 microwire and final sensor electrode.

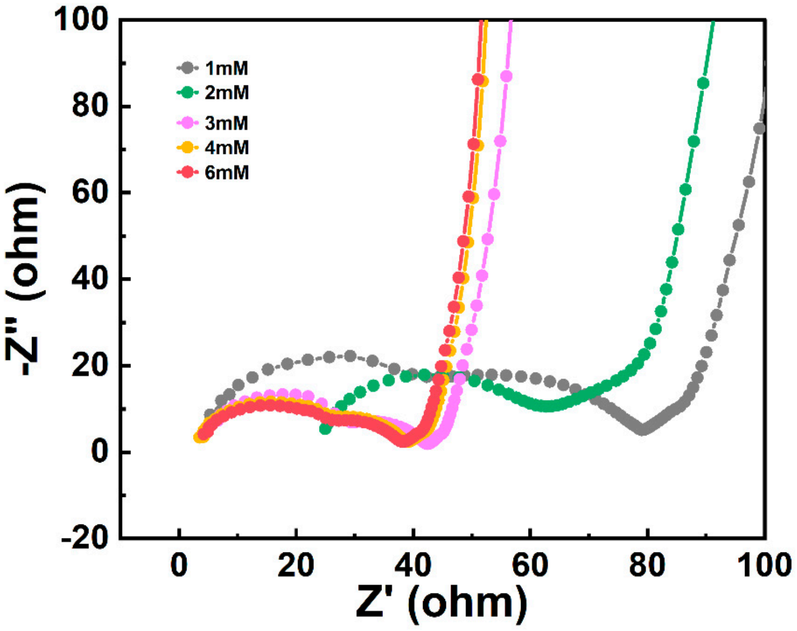

Figure S2 (a) EIS of NW@CuO@Co3O4 microwire in 0.1 M NaOH with different concentration glucose

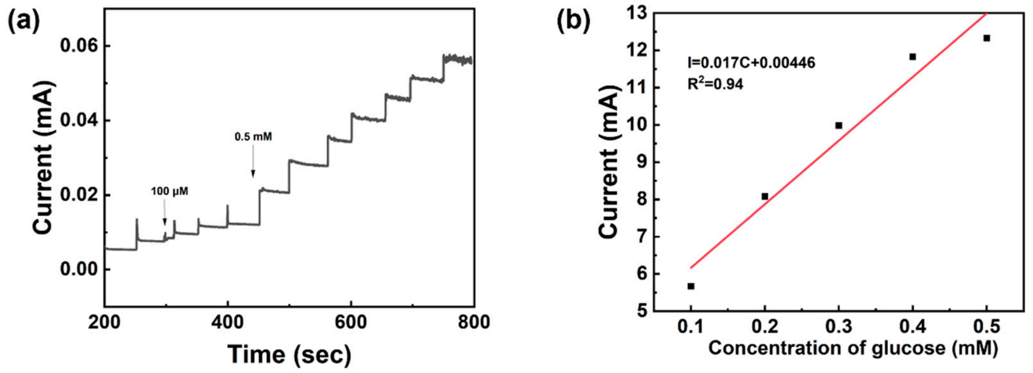

Figure S3 (a) Bare NW Amperometric response curve of adding different concentrations of glucose in 0.1 M NaOH solution in sequence at 0.55V（b）Linear fitting diagram of current

A real sample recovery test was done using 50% (10g/20ml) glucose drinks sold on the market. The experiment is as follows: First, add 20 μL of 50% (10g/20ml, the same below) glucose solution to the electrolyte. Then, add 1 mol·L^{-1} glucose standard solution five times in succession, with 20 μL added each time. Utilize the I-t method for testing and perform a linear fit on the current density and glucose concentration. The glucose concentration was calculated and shown in Table S1 and Figure S4. The recoveries were in the range of 97%–103.5%, illustrating the core-sheath wire electrode has outstanding reliability for glucose determination.

Table S1. Determination of glucose concentration in drinks by NW@CuO@Co_3O_4 electrode

Sample number	Known concentration/mM	Test result/mM	Recovery
1	2.85	2.95	103.5%
2	2.85	2.77	97%
3	2.85	2.71	95%
4	2.85	2.92	102%

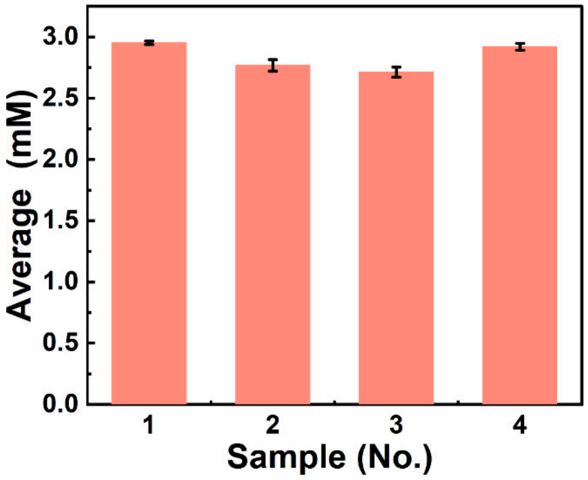

Figure S4 Determination of glucose concentration in drinks by NW@CuO@Co_3O_4 electrode.
